# Snapshot of knowledge and stigma toward mental health disorders and treatment in Spain

**DOI:** 10.3389/fpsyg.2024.1372955

**Published:** 2024-08-30

**Authors:** Andrea Varaona, Rosa M. Molina-Ruiz, Luis Gutiérrez-Rojas, Maria Perez-Páramo, Guillermo Lahera, Carolina Donat-Vargas, Miguel Angel Alvarez-Mon

**Affiliations:** ^1^Deparment of Medicine and Medical Specialties, Faculty of Medicine and Health Sciences, University of Alcala, Alcala de Henares, Spain; ^2^Instituto de Investigación Sanitaria, Hospital Clínico San Carlos, Madrid, Spain; ^3^Department of Psychiatry and Mental Health, Hospital Clínico San Carlos, Madrid, Spain; ^4^Psychiatry Service, Hospital Universitario San Cecilio, Granada, Spain; ^5^Department of Psychiatry and CTS-549 Research Group, Institute of Neurosciences, University of Granada, Granada, Spain; ^6^Medical Department Viatris, Madrid, Spain; ^7^Ramón y Cajal Institute of Sanitary Research (IRYCIS), Madrid, Spain; ^8^Psychiatry Service, University Hospital Principe de Asturias Alcalá de Henares, Madrid, Spain; ^9^Center for Biomedical Research in the Mental Health Network (CIBERSAM), Madrid, Spain; ^10^ISGlobal, Barcelona, Spain; ^11^CIBER Epidemiología y Salud Pública (CIBERESP), Madrid, Spain; ^12^Unit of Cardiovascular and Nutritional Epidemiology, Institute of Environmental Medicine, Karolinska Institute, Stockholm, Sweden; ^13^Department of Psychiatry and Mental Health, Hospital Universitario Infanta Leonor, Madrid, Spain

**Keywords:** public perception, mental illness, stigma, awareness, media

## Abstract

**Background:**

Mental disorders significantly impact individuals and societies globally. Addressing societal stigma is crucial, as it affects help-seeking behaviors and the effectiveness of treatment for mental disorders.

**Objective:**

This study aims to explore the knowledge and perceptions of the Spanish population toward mental disorders and their treatment, assess changes in mental health stigma over time, and examine variations across sociodemographic variables by comparing current results with previous studies.

**Methods:**

A panel of three psychiatrists designed a questionnaire to collect public opinions about various aspects of mental illness in Spain, covering topics such as social perception, diagnostic reliability, causes, symptoms, treatment, suicide, and media influence. The survey, conducted from October to December 2022, involved 1,002 Spanish individuals aged 18–70.

**Results:**

Our results indicated an improved general knowledge of mental health, reduced stigma, and greater acceptance of those affected by these disorders, compared to past research. Almost 80% of the participants have accurate knowledge regarding the complex and multifactorial nature of mental illnesses. Around 90% of the participants share the belief that stigma affects those affected by mental disorders. Psychological and pharmacological treatment are considered to be effective and helpful by more than 70% of the sample. More than 60% of the participants highlighted that suicide needs to be addressed appropriately in the media.

**Conclusion:**

These findings suggest a significant shift in how Spanish society views mental disorders, marking progress over decades of discrimination. Reducing the stigma associated with mental health can encourage individuals to seek assistance without the fear of judgment or discrimination, thereby increasing the likelihood of early intervention and treatment. Open conversations about mental health within families, communities, and workplaces can create a supportive environment that enhances recovery. However, continued efforts and awareness campaigns targeted to broader audiences remain necessary. Responsible media portrayals of mental health, avoiding stigmatizing language or sensationalism, are also essential.

## 1 Introduction

Mental disorders present ongoing challenges to individuals, families, and societies worldwide. They have a significant impact on quality of life and have garnered attention from researchers, clinicians, and policymakers. The World Health Organization estimates that in 2019, 970 million people, or 1 in every 8 individuals, were living with a mental disorder, with depression and anxiety being among the most prevalent^1^ (Auerbach et al., 2018). This emphasizes the need to understand the prevalence, treatment, and societal perceptions surrounding mental health. Studies conducted globally consistently indicate the widespread nature of mental disorders, with the Global Burden of Diseases Study reporting a 48.1% increase in prevalence between 1990 and 2019 (GBD, 2019). A recent study in Spain found a prevalence rate of 13.8% for mental disorders (Maestre-Miquel et al., 2021). Furthermore, disability arising from mental health disorders represents another pivotal dimension to be taken into account. In 2016, mental health disorders accounted for the loss of over 160 million DALYs (Disability-Adjusted Life Years) (Rehm and Shield, 2019). Mental health disorders have also been associated with decreased life expectancy and a deterioration in the quality of life of family members (Harris and Barraclough, 1998; Dey et al., 2012; Roerecke and Rehm, 2014).

Given the burden of mental health disorders, research on risk and protective factors has expanded. Understanding these factors is crucial for comprehending the development, course, and treatment of mental disorders. Various studies highlight the influence of biological, psychological, social, and environmental dimensions on mental health disorders (Hettema et al., 2001; Korkeila et al., 2005; Schiele and Domschke, 2018; Arango et al., 2021). The complexity of mental disorders is reflected in the interplay of these factors. Biological factors, such as genetics and neurochemical imbalances, interact with psychological and social factors, while environmental factors, such as socioeconomic status and exposure to adversity, further contribute to the complexity (Stilo and Murray, 2019; Ortega et al., 2022). The social and environmental dimensions of mental health disorders encompass the beliefs and perceptions held by individuals within a population regarding mental disorders, which in turn influence the developmental trajectory, prognosis, and treatment options available for individuals affected by such disorders. Recognizing this complexity allows healthcare providers to adopt a holistic approach to address mental health, leading to more comprehensive and personalized interventions. By identifying and targeting modifiable risk factors, interventions can be designed to reduce the likelihood of mental disorders.

Understanding mental disorders, along with their causes and risk factors, is essential for combating the stigma associated with mental health (Klin and Lemish, 2008). Mental disorders are treatable and preventable, yet help-seeking is often hindered by stigmatizing attitudes toward mental illness, individuals who suffer from them, and seeking professional help (Schnyder et al., 2018). Stigma toward mental illness and mental health services has been associated with less active help-seeking, and stigma toward psychological and pharmacological treatment can affect therapeutic efficacy and medication adherence (Barnet et al., 2006; Castaldelli-Maia et al., 2011; Nersessova et al., 2019).

Past research in Spain indicates the presence of stigma toward mental illnesses and those who suffer from them. Some studies reveal that the majority of participants perceived individuals with mental health conditions as unpredictable, dangerous, and aggressive (García-Galindo et al., 2012; Ruiz et al., 2012; Mogollón-Rodríguez et al., 2014; Lahera et al., 2019; Zamorano et al., 2023) while other studies reflect the common belief that people with mental illnesses are considered incapable and less responsible, lacking personal autonomy, and facing limitations in the workplace (Aznar-Lou et al., 2016; Lahera et al., 2019). Additionally, there are mixed findings regarding the treatment of mental illnesses, with some indicating that the Spanish population perceived them as less useful due to viewing mental illnesses as chronic conditions (Muñoz et al., 2009; Ruiz et al., 2012), while others showcase the belief in the effectiveness of psychological and psychiatric treatments (Ruiz et al., 2012; Lahera et al., 2019). Addressing the beliefs and perceptions of the population toward mental disorders and their treatment is crucial to tackle stigma, as it greatly impacts self-help seeking and mental health treatment (Lahera et al., 2019).

The current study aims to explore the knowledge and perceptions of the Spanish population toward mental disorders, individuals who suffer them and the treatment of these illnesses. We also aim to assess changes in mental health stigma over time and examine how perceptions and beliefs vary across sociodemographic variables (age, sex, and educational level). However, we will not achieve this objective directly since this study is not longitudinal. Instead, we plan to accomplish it by comparing our results with those of previous studies.

## 2 Materials and methods

An expert panel consisting of three highly esteemed psychiatrists reached a consensus on drafting a questionnaire to achieve the objectives of this study. The process of developing this questionnaire was as follows: the experts agreed on the relevant aspects of mental illness on which they wanted to gather the opinions of the Spanish population. These aspects included social perception and stigma, diagnostic reliability, causes of the illness, symptoms, treatment, suicide, media influence, and sources of information. They then constructed a questionnaire that included questions designed to address the aforementioned topics. The questions were prepared to be relevant, specific, straightforward, understandable to the general population, and free from biases in their wording. Questions that were redundant or did not meet these criteria were discarded. Finally, 13 definitive questions were selected to collect the required information. Additionally, some questions were included to gather sociodemographic data from participants (gender, age, place of residence, number of people living in the household, presence of children in the household, educational level, and current employment status). Finally, a Spanish sociological research company administered a total of 1,002 surveys between October and December 2022. The questionnaire was administered over the phone to Spanish individuals aged 18–70.

Statistical analyses were conducted using Stata. Data analysis was performed using the IBM SPSS Data Collection Base Professional program (v.6.0.1).

This study is compliant with the research ethics principles of the Declaration of Helsinki (seventh revision, 2013). This study did not involve patients, nor did it include any intervention.

## 3 Results

In Table 1, you can observe the distribution by sex, age, education level, and employment status of the participants, as well as the number of children they live with and the region of Spain they reside in.

**Table 1:** Sociodemographic variables from our sample.

Sociodemographic variables	Total
**Sex**	
Men	542 (54.09%)
Women	460 (45.91%)
**Age**	
18–34 years old	123 (12.27%)
35–49 years old	607 (60.58%)
50–70 years old	272 (27.15%)
**Children that live with you**	
Yes	549 (54.79%)
No	453 (45.21%)
Level of education	
Primary education	59 (5.89%)
Secondary/higher education	943 (94.11%)
**Employment status**	
Working or studying	799 (79.82%)
Retired	68 (6.79%)
Unemployed/ERTE	134 (13.39%)
**Province in Spain**	
Andalucía	66 (6.59%)
Aragón	80 (7.98%)
Asturias/Cantabria	20 (2%)
Castilla-La Mancha	84 (8.38%)
Castilla y León	116 (11.58)
Cataluña	25 (2.5%)
Extremadura	181 (18.06%)
Galicia	244 (24.35%)
Comunidad Valenciana	38 (3.79%)
La Rioja/Navarra	14 (1.4%)
Madrid	17 (1.7%)
Murcia	11 (1.1%)
País Vasco	45 (4.49%)
Islas/Ceuta/Melilla	61 (6.09%)

### 3.1 Responses from all participants

When asking participants about the first mental illness that comes to their mind, 47.50% responded mood disorders, while 32.34% indicated that severe and incurable illnesses like schizophrenia first came to their mind. When asked which statement they agreed with the most regarding mental illnesses, half of the participants (51%) agreed that they are subjective and difficult to diagnose, while only 3.19% responded that they are somewhat invented illnesses.

Regarding the causes of psychiatric illnesses, 78.84% responded that the origin of these illnesses is complex and influenced by multiple factors. 10% attributed it to social or genetic origins, while 7% believed the cause of psychiatric illnesses is completely unknown. In terms of individuals with mental illnesses, approximately 59% of participants strongly agreed or agreed that these individuals can lead a normalized life, while 15% held the opposite opinion.

Concerning the stigma surrounding people with mental illnesses, more than half of the sample (51.70%) strongly disagreed that a person with a mental illness should hide it. However, around 7% of respondents believed they should conceal their condition. Furthermore, 97% of participants considered that even today, the stigma surrounding mental illnesses influences seeking help and acts as an obstacle to seeking it.

Regarding treatment, 70% of participants believed that psychotropic medications are often useful and effective, and a slightly higher percentage believed that psychotherapy is usually helpful in most cases. On the contrary, around 20% of participants believed it is better not to take pharmacological treatment due to side effects, or that psychotherapeutic treatment is only worthwhile in specific cases because, despite being effective, it requires a significant time investment.

Regarding the topic of suicide and its portrayal in the media, more than half of the participants considered it important to discuss this topic appropriately. When queried about the appropriate response when someone discloses suicidal ideation or self-harming tendencies, a significant majority of participants advocated for a tactful approach, emphasizing the importance of delicately inquiring about the individual’s emotional wellbeing and gathering relevant information. A minority of participants held the belief that it is better preferable to refrain from asking or to employ humor in an attempt to alleviate the situation and subsequently divert the conversation to a different topic.

Finally, when surveying participants how much they agreed that the National Health Service provides good care for the diagnosis and treatment of mental illnesses, a larger portion of participants (40%) disagreed compared to those who considered the care to be good (26%). When asked about their opinion regarding the care provided by mental health professionals in the National Health Service, almost 70% considered it adequate and good, while 30% evaluated it as inadequate, limited, or insufficient (Table 2).

**Table 2:** Responses from all participants.

When you hear about mental illnesses, what is the first one that comes to mind?	Frequency (percentage) of the total sample
Schizophrenia and related psychotic disorders	324 (32.34%)
Mood disorders (like depression)	476 (47.50%)
Sleep disorders (such as insomnia)	35 (3.49%)
Stress-related disorders (like anxiety)	149 (14.87%)
Others	18 (1.80%)
**Of the following statements about mental illnesses, with which one do you most agree with?**	
Common and easy to treat	270 (26.95%)
Somewhat invented	32 (3.19%)
Subjective, hard to diagnose	511 (51%)
Incurable	189 (18.86%)
**In your opinion, which of the following statements best describes the cause of mental illnesses?**	
Totally unknown	64 (6.39%)
Primarily social	57 (5.69%)
Primarly genetic	41 (4.09%)
Complex and multifactorial	791 (78.94%)
I do not know	49 (4.89%)
**“A person with a mental illness can lead a normal life”**	
Strongly disagree	33 (3.29%)
Somewhat disagree	133 (13.27%)
Neither agree nor disagree	251 (25.05%)
Somewhat agree	434 (43.31%)
Strongly agree	151 (15.07%)
**A person with a mental illness should hide that they have it**	
Strongly disagree	(51.70%)
Somewhat disagree	246 (24.55%)
Neither agree nor disagree	173 (17.27%)
Somewhat agree	(4.59%)
Strongly agree	19 (1.89%)
Do you think the stigma of mental illnesses influences when seeking help	
It definitely influences, it creates shame to admit having a problem.	325 (32.44%)
It does influence, there is a lack of information about mental illnesses.	646 (64.47%)
It does not influence, it’s a thing of the past.	31 (3.09%)
Regarding the treatment of mental illnesses, which of the following statements do you most agree with?	
Psychotropic drugs are addictive and do not cure.	326 (32.53%)
Psychotropic drugs are often helpful and effective.	676 (67.47%)
In terms of treatment of mental illnesses, which of the following statements do you most agree with?	
Psychotherapy is only effective in mild cases.	281 (28.04%).
Psychotherapy is often helpful in most cases.	721 (71.96%)
And for the following statements regarding the treatment of mental illnesses, which one do you most agree with?	
Psychological and pharmacological treatment are effective.	739 (73.75%)
Pharmacological treatment is effective but has side effects. It’s better not to take it.	96 (9.58%)
Psychotherapeutic treatment is effective but requires a significant time investment. It’s worthwhile in specific cases.	125 (12.48%)
None of the two treatments is effective.	42 (4.19%)
**To what extent do you think the topic of suicide should be addresses in the media?**	
Always	203 (20.26%)
Yes, if addressed appropriately	637 (63.57%)
Only for public figures	41 (4.09%)
No, as it can lead to imitation	121 (12.08%)
**If someone tells us they have suicidal or self-harming thoughts, the best course of action is…**	
Not asking.	(8.48%)
Making a joke to relieve tension and change the subject.	34 (3.39%)
Asking delicately and gathering information about their mood.	855 (85.33%)
None of the above. Such cases are extremely rare in developed countries, and there are hardly any suicides.	28 (2.80%)
**The National Health Service provides good care for the diagnosis and treatment of mental illnesses.**	
Strongly disagree	150 (14.97%)
Somewhat disagree	258 (25.75%)
Neither agree nor disagree	329 (32.83%)
Somewhat agree	221 (22.06%)
Strongly agree	44 (4.39%)
**To what extent do you agree with the following statement? “Mental health professionals in the National Health Service provide care that is…**	
Absolutely inadequate	96 (9.58%)
Scarce or insufficient	219 (21.86%)
Adequate	339 (33.83)
Generally good	280 (27.94%)
Excellent	68 (6.79%)

### 3.2 Responses from participants stratified by age

When analyzing by age groups, we observed statistically significant differences among the different groups regarding their opinions on mental disorders. In all three age groups, the majority of participants in each group considered mental illnesses to be subjective and difficult to diagnose. However, it was in the youngest age group where we found the highest percentage of respondents who believed mental illnesses to be incurable.

When participants were asked about individuals with mental illnesses being able to lead a normalized life, we observed agreement among the majority of participants in all three age groups, but the percentage increased as the age of the respondents increased. Therefore, the oldest age group had the highest percentage (approximately 67%) of participants who strongly agreed that people with mental illnesses can lead a normalized life.

We also found statistically significant differences when asking participants about pharmacological treatment for mental illnesses. Although more than half of the participants in each age group believed that psychotropic medications are usually useful and effective, it was the 50–70 age group that had the most positive evaluation of psychotropic medications. When delving further into the treatment of mental illnesses, we observed that the vast majority of participants in each group (between 67 and 82%) perceived both psychological and pharmacological treatments as effective. However, once again, the older age group had a more positive assessment of both treatments.

Regarding the topic of discussing suicide in the media, we also observed statistically significant differences across age groups. The younger participants were more in favor of addressing suicide in the media. Finally, participants in the 35–49 age group were the most hesitant to ask about suicidal thoughts or self-harm tendencies in someone who exhibits them (Table 3).

**Table 3:** Responses from patients stratified by age.

	**18–34 years**	**35–49 years**	**50–70 years**	***p* value**
**When you hear about mental illnesses, what is the first one that comes to mind?**				0.564
Schizophrenia and related psychotic disorders	43 (34.96%)	206 (33.94%)	75 (27.57%)	
Mood disorders (like depression)	55 (44.72%)	275 (45.30%)	146 (53.68%)	
Sleep disorders (such as insomnia)	4 (3.25%)	22 (3.62%)	9 (3.31%)	
Stress-related disorders (like anxiety)	19 (15.44%)	94 (15.49%)	36 (13.24%)	
Others	2 (1.63%)	10 (1.65%)	6 (2.20%)	
**Of the following statements about mental illnesses, with which one do you most agree with?**				0.001
Common and easy to treat	29 (23.58)	155 (25.54%)	86 (31.62%)	
Somewhat invented	9 (7.32%)	23 (3.78%)	0	
Subjective, difficult to diagnose	54 (43.90%)	322 (53.05%)	135 (49.63%)	
Incurable	31 (25.20%)	107 (17.63%)	51 (18.75%)	
**In your opinion, which of the following statements best describes the cause of mental illnesses?**				0.279
Totally unknown	5 (4.06%)	42 (6.92%)	17 (6.25%)	
Primarily social	7 (5.69%)	41 (6.75%)	9 (3.31%)	
Primarily genetic	7 (5.69%)	26 (4.28%)	9 (2.94%)	
Complex and multifactorial	95 (77.24%)	469 (77.27%)	227 (83.46%)	
I do not know	9 (7.32%)	29 (4.78%)	11 (4.04%)	
**A person with a mental illness can lead a normalized life**				0.005
Strongly disagree	8 (6.50%)	20 (3.29%)	5 (1.84%)	
Somewhat disagree	23 (18.70%)	80 (13.18%)	30 (11.02%)	
Neither agree nor disagree	37 (30.08%)	159 (26.20%)	55 (20.22%)	
Somewhat agree	38 (30.9%)	258 (42.50%)	138 (50.74%)	
Strongly agree	17 (13.82%)	90 (14.83%)	44 (16.18%)	
**A person with a mental illness should hide that they have it**				0.169
Strongly disagree	62 (50.41%)	306 (50.41%)	150 (55.15%)	
Somewhat disagree	37 (30.08%)	140 (23.06%)	69 (25.37%)	
Neither agree nor disagree	15 (12.20%)	123 (20.26%)	35 (12.87%)	
Somewhat agree	6 (4.88%)	28 (4.61%)	12 (4.41%)	
Strongly agree	3 (2.43%)	10 (1.65%)	6 (2.20%)	
**Do you think the stigma of mental illnesses influences when seeking help?**				0.628
It definitely influences, it creates shame to admit having a problem	42 (34.15%)	195 (32.13%)	88 (32.35%)	
It does influence, there is a lack of information about mental illnesses	80 (65.04%)	392 (64.58%)	174 (63.97%)	
It does not influence, it’s a thing of the past	1 (0.81%)	20 (3.29%)	10 (3.68%)	
**Regarding the treatment of mental illnesses, which of the following statements do you most agree with?**				0.004
Psychotropic drugs are addictive and do not cure	38 (30.89%)	220 (36.24%)	68 (25%)	
Psychotropic drugs are often helpful and effective	85 (69.11%)	387 (63.76%)	204 (75%)	
**In terms of treatment of mental illnesses, which of the following statements do you most agree with?**				0.191
Psychotherapy is only effective in mild cases	38 (30.89%)	178 (29.32%)	65 (23.90%)	
Psychotherapy is often helpful in most cases	85 (69.11%)	429 (70.68%)	207 (76.10%)	
**And for the following statements regarding the treatment of mental illnesses, which one do you most agree with?**				0.011
Psychological and pharmacological treatment are effective	83 (67.48%)	433 (71.33%)	223 (81.99%)	
Pharmacological treatment is effective but has side effects. It’s better not to take it	17 (13.82%)	61 (10.05%)	18 (6.62%)	
Psychotherapeutic treatment is effective but requires a significant time investment. It’s worthwhile in specific cases	17 (13.82%)	88 (14.50%)	20 (7.35%)	
None of the two treatments is effective	6 (4.88%)	25 (4.12%)	11 (4.04%)	
**To what extent do you think the topic of suicide should be addressed in the media?**				0.000
Always	42 (34.15%)	116 (19.11%)	45 (16.54%)	
Yes, if addressed appropriately	69 (56.10%)	372 (61.29%)	196 (72.06%)	
Only for public figures	4(3.25%)	32(5.27%)	5(1.84%)	
No, as it can lead to imitation	8 (6.50%)	87 (14.33%)	26 (9.56%)	
**If someone tells us they have suicidal or self-harming thoughts, the best course of action is…**				0.043
Not asking	8 (6.50%)	58 (9.56%)	19 (6.99%)	
Making a joke to relieve tension and change the subject	4 (3.25%)	28 (4.61%)	2 (0.73%)	
Asking delicately and gathering information about their mood	107 (87%)	502 (82.70%)	246 (90.44%)	
None of the above. Such cases are extremely rare in developed countries, and there are hardly any suicides.	4 (3.25%)	19 (3.13%)	5 (1.84%)	
**The National Health Service provides good care for the diagnosis and treatment of mental illnesses**				0.136
Strongly disagree	15 (12.20%)	104 (17.13%)	31 (11.40%)	
Somewhat disagree	37 (30.08%)	146 (24.05%)	75 (27.57%)	
Neither agree nor disagree	39 (31.71%)	207 (34.10%)	83 (30.51%)	
Somewhat agree	24 (19.51%)	125 (20.6%)	72 (26.47%)	
Strongly agree	8 (6.50%)	25 (4.12%)	11 (4.04%)	
**To what extent do you agree with the following statement? “Mental health professionals in the National Health Service provide care that is…**				0.078
Absolutely inadequate	15 (12.20%)	61 (10.05%)	20 (7.35%)	
Scarce or insufficient	36 (29.27%)	126 (20.76%)	57 (20.96%)	
Adequate	29 (23.58%)	221 (36.41%)	89 (32.72%)	
Generally good	37 (30.08%)	158 (26.03%)	85 (31.25%)	
Excellent	6 (4.87%)	41 (6.75%)	21 (7.72%)	

### 3.3 Responses from participants stratified by gender

When analyzing the results by gender, we found that women were more in agreement than men regarding the idea that individuals with a mental illness can lead a normalized life. Approximately 62% of women strongly agreed or agreed with this notion, while the percentage of men who agreed with this statement was lower at 55%. Secondly, we observed statistically significant differences between genders in terms of how to react when learning that someone has suicidal thoughts or self-harm tendencies. Finally, we found statistically significant differences in the perception of mental health professionals in the National Health Service. Men exhibited a more favorable evaluation of mental health professionals (Table 4).

**Table 4:** Responses from participants stratified by gender.

	Men	Women	*P-*value
**When you hear about mental illnesses, what is the first one that comes to mind?**			0.264
Schizophrenia and related psychotic disorders	172 (31.73%)	152 (33.04%)	
Mood disorders (like depression)	251 (46.31%)	225 (48.91%)	
Sleep disorders (such as insomnia)	25 (4.61%)	10 (2.17%)	
Stress-related disorders (like anxiety)	83 (15.31%)	66 (14.35%)	
Others	11 (2.03%)	7 (1.52%)	
**Of the following statements about mental illnesses, which one do you most agree with?**			0.369
Common and easy to treat	143 (26.38%)	127 (27.61%)	
Somewhat invented	22 (4.06%)	10 (2.17%)	
Subjective, difficult to diagnose	278 (51.29%)	233 (50.65%)	
Incurable	99 (18.27%)	90 (19.57%)	
**In your opinion, which of the following statements best describes the cause of mental illnesses?**			0.060
Totally unknown	30 (5.54%)	34 (7.39%)	
Primarily social	31 (5.72%)	26 (5.65%)	
Primarily genetic	30 (5.54%)	11 (2.39%)	
Complex and multifactorial	429 (79.15%)	362 (78.70%)	
I do not know	22 (4.06%)	27 (5.87%)	
**A person with a mental illness can lead a normalized life**			0.007
Strongly disagree	17 (3.14%)	16 (3.48%)	
Somewhat disagree	88 (16.24%)	45 (9.78%)	
Neither agree nor disagree	139 (25.65%)	112 (24.35%)	
Somewhat agree	231 (42.62%)	203 (44.13%)	
Strongly agree	67 (12.36%)	84 (18.26%)	
**A person with a mental illness should hide that they have it**			0.125
Strongly disagree	266 (49.08%)	252 (54.78%)	
Somewhat disagree	149 (27.49%)	97 (21.09%)	
Neither agree nor disagree	90 (16.61%)	83 (18.04%)	
Somewhat agree	28 (5.17%)	18 (3.91%)	
Strongly agree	9 (1.66%)	10 (2.17%)	
**Do you think the stigma of mental illnesses influences when seeking help?**			0.825
It definitely influences, it creates shame to admit having a problem	172 (31.73%)	153 (33.26%)	
It does influence, there is a lack of information about mental illnesses	354 (65.31%)	292 (63.48%)	
It does not influence, it’s a thing of the past	16 (2.95%)	15 (3.26%)	
**Regarding the treatment of mental illnesses, which of the following statements do you most agree with?**			0.963
Psychotropic drugs are addictive and do not cure	176 (32.47%)	150 (32.61%)	
Psychotropic drugs are often helpful and effective	366 (67.53%)	310 (67.39%)	
**In terms of treatment of mental illnesses, which of the following statements do you most agree with?**			1.000
Psychotherapy is only effective in mild cases	152 (28.04%)	129 (28.04%)	
Psychotherapy is often helpful in most cases	390 (71.96%)	331 (71.96%)	
**And for the following statements regarding the treatment of mental illnesses, which one do you most agree with?**			0.276
Psychological and pharmacological treatment are effective	389 (71.77%)	350 (76.09%)	
Pharmacological treatment is effective but has side effects. It’s better not to take it	56 (10.33%)	40 (8.70%)	
Psychotherapeutic treatment is effective but requires a significant time investment. It’s worthwhile in specific cases	76 (14.02%)	49 (10.65%)	
None of the two treatments is effective	21 (3.87%)	21 (4.57%)	
**To what extent do you believe the topic of suicide should be addressed in the media?**			0.683
Always	103 (19%)	100 (1.74%)	
Yes, if it is addressed appropriately	347 (64.02%)	290 (63.04%)	
Only for public figures	24 (4.43%)	17 (3.70%)	
No, as it can lead to imitation	68 (12.55%)	53 (11.52%)	
**If someone tells us they have thoughts of suicide or self-harm, the best course of action is…**			0.034
Not asking	48 (8.86%)	37 (8.04%)	
Making a joke to relieve tension and change the subject	26 (4.80%)	8 (1.74%)	
Asking delicately and gathering information about their mood	456 (84.13%)	399 (86.74%)	
None of the above. Such cases are extremely rare in developed countries, and there are hardly any suicides.	12 (2.21%)	16 (3.48%)	
**The National Health Service provides good care for the diagnosis and treatment of mental illnesses**			0.129
Strongly disagree	75 (13.84%)	75 (16.30%)	
Somewhat disagree	128 (23.62%)	130 (28.26%)	
Neither agree nor disagree	196 (36.16%)	133 (28.91%)	
Somewhat agree	120 (22.14%)	101 (21.96%)	
Strongly agree	23 (4.24%)	21 (4.57%)	
**To what extent do you agree with the following statement? “Mental health professionals in the National Health Service provide care that is…**			0.013
Absolutely inadequate	43 (7.93%)	53 (11.52%)	
Scarce or insufficient	103 (19%)	116 (25.22%)	
Adequate	203 (37.45%)	136 (29.57%)	
Generally good	155 (28.60%)	125 (27.17%)	
Excellent	38 (7.01%)	30 (6.52%)	

### 3.4 Responses from participants stratified by educational level

When analyzing the results according to participants’ educational level, we found that those with higher levels of education more strongly believed that mental illnesses are subjective and difficult to diagnose compared to those with lower levels of education. Additionally, individuals with higher levels of education were more likely to respond that mental illnesses are common and easily treatable. On the other hand, participants with lower levels of education were three times more likely to consider these illnesses as somewhat invented compared to those with higher levels of education (Table 5).

**Table 5:** Responses from participants stratified by educational level.

	High school	Higher education	*P-*value
**When you hear about mental illnesses, what is the first one that comes to mind?**			0.939
Schizophrenia and related psychotic disorders	19 (32.20%)	305 (32.34%)	
Mood disorders (like depression)	26 (44.07%)	450 (47.72%)	
Sleep disorders (such as insomnia)	3 (5.08%)	32 (3.39%)	
Stress-related disorders (like anxiety)	10 (16.95%)	139 (14.74%)	
Others	1 (1.69%)	17 (1.80%)	
**Of the following statements about mental illnesses, which one do you most agree with?**			0.041
Common and easy to treat	12 (20.34%)	258 (27.36%)	
Somewhat invented	5 (8.47%)	27 (2.86%)	
Subjective, difficult to diagnose	27 (45.76%)	484 (51.33%)	
Incurable	15 (25.42%)	174 (18.45%)	
**In your opinion, which of the following statements best describes the cause of mental illnesses?**			0.023
Totally unknown	2 (3.39%)	62 (6.57%)	
Primarily social	7 (11.86%)	50 (5.30%)	
Primarily genetic	0	41 (4.35%)	
Complex and multifactorial	44 (74.58%)	747 (79.22%)	
I do not know	6 (10.17%)	43 (4.56%)	
**A person with a mental illness can lead a normalized life**			0.347
Strongly disagree	3 (5.08%)	30 (3.18%)	
Somewhat disagree	9 (15.25%)	124 (13.15%)	
Neither agree nor disagree	20 (33.90%)	231 (24.50%)	
Somewhat agree	20 (33.90%)	414 (43.90%)	
Strongly agree	7 (11.86%)	144 (15.27%)	
**A person with a mental illness should hide that they have it**			0.416
Strongly disagree	24 (40.68%)	494 (52.39%)	
Somewhat disagree	16 (27.12%)	230 (24.39%)	
Neither agree nor disagree	14 (23.73%)	159 (16.86%)	
Somewhat agree	3 (5.08%)	43 (4.56%)	
Strongly agree	2 (3.39%)	17 (1.80%)	
**Do you think the stigma of mental illnesses influences when seeking help?**			0.639
It definitely influences, it creates shame to admit having a problem	17 (28.81%)	308 (32.66%)	
It does influence, there is a lack of information about mental illnesses	41 (69.49%)	605 (64.16%)	
It does not influence, it’s a thing of the past	1 (1.69%)	30 (3.18%)	
**Regarding the treatment of mental illnesses, which of the following statements do you most agree with?**			0.169
Psychotropic drugs are addictive and do not cure	24 (40.68%)	302 (32.03%)	
Psychotropic drugs are often helpful and effective	35 (59.32%)	641 (67.97%)	
**In terms of treatment of mental illnesses, which of the following statements do you most agree with?**			0.664
Psychotherapy is only effective in mild cases	18 (30.51%)	263 (27.89%)	
Psychotherapy is often helpful in most cases	41 (69.49%)	680 (72.11%)	
**Of the following statements regarding the treatment of mental illnesses, which one do you most agree with?**			0.636
Psychological and pharmacological treatment are effective	40 (67.80%)	699 (74.13%)	
Pharmacological treatment is effective but has side effects. It’s better not to take it	7 (11.86%)	89 (9.44%)	
Psychotherapeutic treatment is effective but requires a significant time investment. It’s worthwhile in specific cases	8 (13.56%)	117 (12.41%)	
None of the two treatments is effective	4 (6.78%)	38 (4.03%)	
**To what extent do you believe the topic of suicide should be addressed in the media?**			0.021
Always	11 (18.64%)	192 (20.36%)	
Yes, if it is addressed appropriately	35 (59.32%)	602 (63.84%)	
Only for public figures	7 (11.86%)	34 (3.61%)	
No, as it can lead to imitation	6 (10.17%)	115 (12.20%)	
**If someone tells us they have suicidal or self-harm thoughts, the best course of action is…**			0.791
Not asking	7 (11.86%)	78 (8.27%)	
Making a joke to relieve tension and change the subject	2 (3.39%)	32 (3.39%)	
Asking delicately and gathering information about their mood	48 (81.36%)	807 (85.58%)	
None of the above. Such cases are extremely rare in developed countries, and there are hardly any suicides.	2 (3.39%)	26 (2.76%)	
**The National Health Service provides good care for the diagnosis and treatment of mental illnesses**			0.299
Strongly disagree	4 (6.78%)	146 (15.48%)	
Somewhat disagree	15 (25.42%)	243 (25.77%)	
Neither agree nor disagree	24 (40.68%)	305 (32.34%)	
Somewhat agree	12 (20.34%)	209 (22.16%)	
Strongly agree	4 (6.78%)	40 (4.24%)	
**To what extent do you agree with the following statement? “Mental health professionals in the National Health Service provide care that is…**			0.494
Absolutely inadequate	4 (6.78%)	92 (9.76%)	
Scarce or insufficient	9 (15.25%)	210 (22.27%)	
Adequate	25 (42.37%)	314 (33.30%)	
Generally good	16 (27.12%)	264 (28%)	
Excellent	5 (8.47%)	63 (6.68%)	

Regarding the origin of mental illnesses, almost twice as many participants with higher levels of education, compared to those with lower levels, believed that the cause of these illnesses is unknown. Conversely, twice as many participants with lower levels of education believed that these illnesses have a primarily social origin compared to those with higher levels of education. However, a higher percentage of participants with higher levels of education (79.22%) stated that the origin of mental illnesses is complex and influenced by multiple factors.

Lastly, participants with lower levels of education were less likely to believe that the media should discuss suicide appropriately. Three times as many participants in this group suggested that it is better to talk about this topic only when it concerns public figures, compared to those with higher levels of education.

## 4 Discussion

To our knowledge, this is the first study to address the beliefs and perceptions of people in Spain regarding mental disorders in the aftermath of the pandemic, which has marked a significant milestone in the overall understanding of mental health, including their causes, diagnosis, and treatment, as well as their attitudes toward suicidal and self-harm thoughts and how this matter should be displayed in media.

Results from this investigation show that participants’ knowledge about mental disorders has increased and their consideration toward people who suffer mental illness, and the treatment of mental disorders has become more positive. One major finding is that most of the participants attribute that the origin of mental disorders is complex and it derives from a combination of psychological, biological, and social factors, showing a more accurate conception of mental disorders than in past research conducted in Spain with Spanish medical students (Failde et al., 2014). In a previous study conducted with the general population in Spain, participants displayed less knowledge regarding the cause of mental disorders, where many participants attributed that people who suffer mental disorders have personal control over the cause of their condition (Crespo et al., 2008). In a different study, the vast majority of participants thought that the causes of schizophrenia and bipolar disorder were changes in brain biology, genetics or drug use, while only a minority took into consideration psychosocial factors such as stress, child trauma, or problems in birth or pregnancy (Ruiz et al., 2012).

A second major finding in our study is that more than half of the participants think that a person with a mental disorder can live a normalized life and that they should not hide that they suffer a mental disorder. This result shows that stigma regarding mental disorders has diminished. These results are optimistic as previous research have found high levels of mental health stigma in Spain. In a study conducted in 2020, Spain was the country with higher levels of Stigma, compared to Canada and Russia. In this study, Spanish students showed a higher rate of stereotypes and social distance intentions toward people who suffer mental disorders, where men showed higher levels of stigma than women (Gallego et al., 2020). In a more recent study with university students, 91% of the participants thought that mental illnesses interfere significantly, inhibiting normal life (Ruiz et al., 2022). Our results are in line with the results of a study conducted in 2022, where only a small percentage of the participants thought that people with mental disorders should be separated from the community or perceived them as dangerous or people that should be avoided (Ruiz et al., 2022). Our results shed light on a significant shift in societal attitudes toward individuals with mental disorders, showing a notable increase in the positive consideration and acceptance of people with mental disorders, accompanied by a growing desire to integrate them into the broader community. Unlike the past, where stigma and discrimination were higher, there is now a greater recognition and empathy of the challenges faced by individuals with mental disorders.

Several factors may have contributed to this change in the perception of people with mental disorders. Efforts to raise awareness about mental health, including campaigns, educational programs, and media representation directed to raise awareness about mental health have played a vital role in combating stigma. In the past years, several campaigns and initiatives have helped challenge stereotypes and normalize conversations about mental health (Evans-Lacko et al., 2014; Mehta et al., 2015). On the other hand, the COVID-19 pandemic has had a profound impact on the conversation about mental health (Javakhishvili et al., 2022). The mortality of those infected by the virus, the measures taken to contain the spread of the virus, such as lockdowns, social distancing and restrictions, and the economic instability caused by the decreases in economic activities, have brought a complex set of challenges that have magnified existing mental health issues and brought new ones to the forefront (Santomauro et al., 2021). As Covid-19 was a crisis that affected everyone in multiple ways, this may have compelled individuals to openly acknowledge and address their mental health struggles. The bravery of individuals recognizing and sharing their personal experiences with mental disorders may have prompted a collective recognition of the importance of mental well-being and provided powerful insights for other people who face mental illness. Additionally, advocacy movements led by individuals and organizations have effectively pushed for several policy changes, increased resources, and improved mental health services, ultimately reducing the associated stigma (Sampogna et al., 2017; Henderson et al., 2020). On the other hand, specifically in Spain, prominent figures and institutions have advocated for greater attention to mental well-being, which has effectively gathered the attention of the population. For instance, two of the most sold books in Spain in the last years were focused on self -help and mental health and were written by psychiatrist Marian Rojas Estapé.^2^ This shows that population is more interested in learning about mental health and addressing their personal struggles with self-help books.

Gaining knowledge about the causes of mental disorders helps dispel the myth that they are solely the result of personal weakness or character flaws. In a study conducted in France, schizophrenia was seen as a mental disorder caused majorly by biological and genetic factors and therefore required the help of psychiatrists and psychotropic medication. On the other side, depression was seen as a consequence of stress and was considered to have a better prognosis, where general practitioners, psychotherapy and social support were considered to be the most appropriate treatment (Angermeyer et al., 2013). Similar results were observed in a different study conducted within the general population in Germany, where the cause of depression was mostly attributed to experiencing psychological and social stress, while biological factors were most frequently considered to be the cause of schizophrenia. Nevertheless, participants most frequently reacted with a desire to help toward both people who suffer depression and schizophrenia, and they were both viewed as having a good prognosis with treatment (Angermeyer and Matschinger, 2003). Recognizing that mental disorders are complex conditions influenced by a combination of genetic, biological, environmental, and psychosocial factors encourages a shift from judgment to support, thereby reducing the stigma surrounding mental health.

Moreover, around 70% of the participants think of pharmacological and psychological treatment as useful and effective when addressing mental illness. Several past investigations with patients and the general population have shown that perceived pharmacological treatment for mental disorders as addictive and that rejection of medication was a way of not being labeled as mentally ill or as a weak person who is unable to deal with personal struggles (Comas and Alvarez, 2004; Khan et al., 2007; Prins et al., 2008; Malpass et al., 2009). More than a decade ago, a study showed that people in Spain thought that antidepressants caused dependency and therefore, rejected pharmacological treatment (Comas and Alvarez, 2004). A different study in Spain showed that psychological and pharmacological treatments were perceived as useful by the majority of participants and were considered to be effective. Nevertheless, a large number of participants agreed that only people who show symptoms require mental health treatment, and that psychological treatment lasts a lifetime, which is harmful for the patient (Ruiz et al., 2012). Our results show that consideration of pharmacological and psychological treatment has become more positive in the past years, with more people being aware and certain of their utility and efficacy (Watson and Beshai, 2021). Positive consideration of psychological and pharmacological treatment plays a pivotal role in promoting mental well-being and improving the quality of life for individuals with mental health struggles. By embracing a positive attitude toward these treatments, individuals are more likely to seek help without hesitation, reduce self-stigma, and overcome barriers to treatment, which result in an improved overall well-being and a higher likelihood of successful recovery for those who suffer mental illness.

With regards to suicide, our results show that people have accurate knowledge on how suicide should be addressed in an individual and collective manner. More than 80% of our participants answered that if someone shares suicidal or self-harm thoughts or concerns, the best way to handle it is asking kindly and gathering more information about that person’s mood. Only 3% thought that suicide is extremely rare in developed countries and only 4% thought that the best way to react if someone shares suicidal or self-harm concerns is to make jokes and change subject. In Spain, several suicide campaigns have been created and implemented to equip the population with knowledge and resources to handle personal suicidal or self-harm thoughts, as well as supporting those around them facing similar struggles. To name some of them, “Teléfono de la Esperanza” (Hope Helpline) is a helpline that offers emotional support and intervention for individuals in crisis. Their campaign encourages open conversations about suicide. Mass media has also played a crucial role in suicide prevention and management. For instance, last year, “Radio Televisión Espanola”” (RTVE, Spanish Radio and Television), one of the most important news media in Spain, broadcasted and promoted a campaign called “Me, too,” aimed to share the personal stories of famous figures in Spain who suffer mental struggles.^3^ In our study, around 60% of our participants agreed that suicide should be adequately addressed in media, which shows that our society is more open about these conversations and aware of the importance of having such spaces that contribute to gathering knowledge and resources to better manage suicide.

Furthermore, media coverage of suicide is important as suicide is a leading cause of premature death. The fact that people are aware that suicide is a topic that should be handled in an appropriate way by media is crucial as, if not doing so, several risks can arise (Guinovart et al., 2023). Firstly, sensationalizing suicide can lead to a phenomenon known as suicide contagion or the “copycat effect.” (Sudak and Sudak, 2005; VanderWeele et al., 2019). Inappropriate media coverage can also contribute to increase stigma surrounding mental health and suicide, perpetuating misconceptions, and decreasing discussions about prevention. On the other hand, silence may not be an effective way to handle suicide, as silence and avoidance can perpetuate stigma surrounding mental health and create barriers to help-seeking behavior (Barnet et al., 2006). Open and responsible communication can be beneficial. In addition, responsible reporting and communication guidelines have been developed to promote accurate, compassionate, and prevention-focused discussions about suicide in the media (World Health Organization & International Association for Suicide Prevention, 2017).

A limitation of our study is the absence of a preliminary testing phase for the questionnaire in a smaller sample before its administration to the study participants. While the questions were meticulously formulated by a committee of experts with extensive experience in mental health, the lack of a pilot test introduces an element of uncertainty regarding the clarity, interpretability, and potential ambiguity of certain items. A preliminary test would have allowed us to identify and rectify any ambiguities with the wording of questions, ensuring a more refined instrument for data collection. The omission of this step may have implications for the reliability and validity of the responses obtained, as participants’ interpretations of certain items could vary. Despite this limitation, we aimed to mitigate potential issues by having the expert committee complete the questionnaire, but we acknowledge that a dedicated pilot phase would have offered a more robust foundation for the study. Another noteworthy limitation pertains to the geographical distribution of our participants, with a predominant representation from Castilla y León, Extremadura, and Galicia. This regional concentration may limit the generalizability of our findings to the entire Spanish population, as regional variations in attitudes and perceptions toward mental health could impact the broader applicability of our results. Caution should be exercised when extrapolating our conclusions to a national level, and future research endeavors should strive for a more geographically diverse sample to enhance the external validity of the study. Finally, it is important to keep in mind that the present work may vary with respect to works carried out in the past in methodology and in the historical influences received.

In conclusion, our results are optimistic and somehow indicate that in recent years there has been a significant transformation in how Spanish society perceives mental disorders, leaving behind decades of discrimination, social exclusion, and inadequate care (Sajatovic et al., 2008; Aydemir and Akkaya, 2011; Suto et al., 2012; Hawke et al., 2013). The reduction of mental health stigma can bring numerous benefits. It may encourage individuals to seek help and support without fear of judgment or discrimination, facilitating early intervention and treatment (Coppens et al., 2013). It may also promote open conversations about mental health within families, communities, and workplaces, fostering a supportive environment that may more likely improve recovery. However, it is essential to continue promoting a better comprehension of mental disorders. Education and awareness campaigns should be expanded to reach broader audiences, targeting schools, workplaces, and communities. Media outlets should continue to portray mental health responsibly, avoiding stigmatizing language or sensationalism.

## Data Availability

The raw data supporting the conclusions of this article will be made available by the authors, without undue reservation.

## Footnotes

^1^https://www.who.int/news-room/fact-sheets/detail/mental-disorders

^2^https://www.abc.es/cultura/libros/abci-libro-autoayuda-best-seller-rivaliza-ventas-premio-planeta-201901302043_noticia.html

^3^https://www.rtve.es/rtve/20220908/rtve-lanza-campana-prevencion-sucidio-primera-vez-historia/2401332.shtml
