# Corrigendum: Snapshot of knowledge and stigma toward mental health disorders and treatment in Spain

**DOI:** 10.3389/fpsyg.2025.1616178

**Published:** 2025-05-27

**Authors:** Andrea Varaona, Rosa M. Molina-Ruiz, Luis Gutiérrez-Rojas, Maria Perez-Páramo, Guillermo Lahera, Carolina Donat-Vargas, Miguel Angel Alvarez-Mon

**Affiliations:** ^1^Deparment of Medicine and Medical Specialties, Faculty of Medicine and Health Sciences, University of Alcala, Alcala de Henares, Spain; ^2^Instituto de Investigación Sanitaria, Hospital Clínico San Carlos, Madrid, Spain; ^3^Department of Psychiatry and Mental Health, Hospital Clínico San Carlos, Madrid, Spain; ^4^Psychiatry Service, Hospital Universitario San Cecilio, Granada, Spain; ^5^Department of Psychiatry and CTS-549 Research Group, Institute of Neurosciences, University of Granada, Granada, Spain; ^6^Medical Department Viatris, Madrid, Spain; ^7^Ramón y Cajal Institute of Sanitary Research (IRYCIS), Madrid, Spain; ^8^Psychiatry Service, University Hospital Principe de Asturias Alcalá de Henares, Madrid, Spain; ^9^Center for Biomedical Research in the Mental Health Network (CIBERSAM), Madrid, Spain; ^10^ISGlobal, Barcelona, Spain; ^11^CIBER Epidemiología y Salud Pública (CIBERESP), Madrid, Spain; ^12^Unit of Cardiovascular and Nutritional Epidemiology, Institute of Environmental Medicine, Karolinska Institute, Stockholm, Sweden; ^13^Department of Psychiatry and Mental Health, Hospital Universitario Infanta Leonor, Madrid, Spain

**Keywords:** public perception, mental illness, stigma, awareness, media

In the published article, there was an error in affiliation 6. Instead of “Medical Department, Madrid, Spain,” it should be “Medical Department Viatris, Madrid, Spain.”

Additionally, in the published article, there was an error in the **Funding statement**. This statement originally stated: “The author(s) declare financial support was received for the research, authorship, and/or publication of this article. This research was funded by Viatris. The funder was not involved in the study design, collection, analysis, interpretation of data, the writing of this article, or the decision to submit it for publication.” The correct Funding statement appears below.

